# Prevalence of clinical malaria and household characteristics of patients in tribal districts of Pakistan

**DOI:** 10.1371/journal.pntd.0009371

**Published:** 2021-05-03

**Authors:** Asad Mustafa Karim, Muhammad Yasir, Tanveer Ali, Sumera Kausar Malik, Irfan Ullah, Naveeda Akhtar Qureshi, Huang Yuanting, Esam I. Azhar, Hyung Jong Jin

**Affiliations:** 1 Department of Bioscience and Biotechnology, The University of Suwon, Hwaseong City, Gyeonggi-do, Republic of Korea; 2 Special Infectious Agents Unit, King Fahd Medical Research Center, King Abdulaziz University, Jeddah, Saudi Arabia; 3 Medical Laboratory Technology Department, Faculty of Applied Medical Sciences, King Abdulaziz University, Jeddah, Saudi Arabia; 4 Department of Microbiology and Molecular Genetics, University of the Punjab, Pakistan; 5 Department of Microbiology, Kohat University of Science and Technology, Kohat, Pakistan; 6 Department of Internal Medicine, Section of Infectious Diseases, Yale University School of Medicine, New Haven, United States of America; 7 Department of Animal Sciences, Quaid-e-Azam University Islamabad, Islamabad, Pakistan; Centre hospitalier de Cayenne, FRANCE

## Abstract

**Background:**

Malaria, disproportionately affects poor people more than any other disease of public health concern in developing countries. In resource-constrained environments, monitoring the occurrence of malaria is essential for the success of national malaria control programs. Militancy and military conflicts have been a major challenge in monitoring the incidence and controlling malaria and other emerging infectious diseases. The conflicts and instability in Afghanistan have resulted in the migration of refugees into the war-torn tribal districts of Pakistan’s Khyber Pakhtunkhwa (KPK) province and the possible introduction of many contagious epidemics. Although malaria is very common in all tribal districts, molecular, clinical and epidemiological data are scarce in these high-burden districts. Therefore, for the proper surveillance, detection, and control of malaria, obtaining and analyzing reliable data in these districts is essential.

**Methodology/Principal findings:**

All 1,127 malaria-suspected patients were sampled within the transmission season in the tribal districts of KPK province between March 2016 to December 2018. After a detailed demographic and clinical investigation of malaria-suspected patients, the data were recorded. The data of the control group was collected simultaneously at the same site. They were considered as uncomplicated cases for statistical analyses. Blood samples were collected from malaria-suspected patients for the detection of *Plasmodium* species using microscopy and nested PCR (nPCR). Microscopy and nPCR examination detected 78% (n = 882) and 38% (n = 429) *Plasmodium*-positive patients, respectively. Among1,127 of 429nPCR detected cases with both species of malaria, the frequency of complications was as follows: anemia (n = 71; 16.5%), decompensated shock (n = 40; 9%), hyperpyrexia (n = 117; 27%), hyperparasitaemia (n = 49; 11%) hypoglycemia (n = 45; 10.5%), jaundice (n = 54; 13%), multiple convulsions (n = 37; 9%), and petechia (n = 16; 4%). We observed that 37% (n = 157 out of 429) of those patients infected by both *Plasmodium* species were children between the ages of 1 and 15 years old. The results revealed that Bajaur (24%), Kurram (20%), and Khyber (18%) districtshada higher proportion of *P*. *vivax* than *P*. *falciparum* cases. Most of the malaria cases were males (74%). Patients infected by both *Plasmodium* species tended to less commonly have received formal education and ownership of wealth indicators (e.g., fridge, TV set) was lower.

**Conclusions/Significance:**

Malaria in tribal districts of the KPK province largely affects young males. *P*. *vivax* is a major contributor to the spread of malaria in the area, including severe malaria. We observed a high prevalence of *P*. *vivax* in the Bajaur district. Children were the susceptible population to malaria infections whereas they were the least expected to use satisfactory prevention strategies. A higher level of education, a possession of TV sets, the use of bed nets, the use of repellent fluids, and fridges were all associated with protection from malaria. An increased investment in socio-economic development, a strong health infrastructure, and malaria education are key interventions to reduce malaria in the tribal districts.

## Author summary

The malaria epidemic and endemic in tribal areas of KPK, Pakistan is a present and constant threat to public health. It has been a great challenge in monitoring the incidence of malaria in tribal districts of KPK because of the military conflict. We report an epidemiological, clinical, and household assessment of malaria endemicity in the six tribal districts of KPK, Pakistan, which is the most neglected area due to militancy and war in Afghanistan. The study sample comprised of1,127malaria-suspectedpatients. Their blood samples were collected for microscopy, nPCR and biochemical analysis. Of the samples collected, 78% were identified as *Plasmodium*-positive by microscopic analysis, whereas, 38% were determined as *Plasmodium*-positive by nPCR. Then PCR analysis detected 87% (n = 373 out of 429) and 13% (n = 56 out of 429) *P*. *vivax* and *P*. *falciparum* cases, respectively. Cases were noticeably higher in males (74%). We found poor hygiene conditions in all the districts we examined, probably caused by low socioeconomics settings. Hyperpyrexia (27%) was the prominent clinical feature among all the cases infected by both *P*. *vivax* and *P*. *falciparum*. Our findings have substantial implications for both malaria control and public health in the tribal districts of Pakistan.

## Introduction

Malaria is a mosquito-borne parasitic infectious disease of tropical and subtropical regions worldwide [1]. It imposes greatest health and socioeconomic burden in these regions of the world. According to the World Health Organization (WHO), in their latest malaria report, 2018, there were approximately 228 million malarial cases with 405, 000 deaths (416,000 in 2017) worldwide [2]. Moreover, in 2018, most malaria cases were reported by the WHO Regional Office for Africa (213 million or 93%), followed by the WHO Regional Office for South-East Asia (3.4%) and thirdly, the WHO Regional Office for the Eastern Mediterranean (2.1%) [2]. Five *Plasmodium* species which include *Plasmodium falciparum (P*. *falciparum)*, *P*. *vivax*, *P*. *malariae*, *P*. *ovale*, *and P*. *knowlesi* are responsible for introducing malaria in human beings [3]. However, *P*. *falciparum* is the highest prevalent parasite in the African region accounting for 99.7% malarial cases in 2018 and 71% in the Eastern Mediterranean [2]. *P*. *vivax* is 53% prevalent globally, with the highest cases (47%) being in India (South-East Asia region), while 11% and 8% of *vivax* malaria cases occurred in Afghanistan and Pakistan (Eastern Mediterranean region), respectively [2,4]. Despite the recent progress towards the eradication of malaria, vaccination and anti-microbial therapies, the control of malaria infections is still challenging.

Pakistan remains one of the highest burden-sharing countries in the fight against malaria with an estimated 1 million cases annually [4,5]. According to WHO, currently *P*. *vivax* malaria is 84% prevalent in Pakistan while 14.9% and 1.1% cases are accounted for by *P*. *falciparum* and mixed cases (double infections with *P*. *vivax* and *P*. *falciparum*), respectively [2]. Tribal districts (previously called FATA: Federally Administered Tribal Areas) of KPK being the most impoverished and extremely under developed areas in Pakistan have the highest malaria burden due to the large number of Afghan refugees and internally displaced persons (IDPs) [6–8].

The Afghan-Soviet war predominantly annihilated overall public health especially on the malaria vector control programs in Afghanistan [9]. The collapse of the vector control programs have resulted in the re-emergence of malaria and turned it into a malaria endemic zone [10]. Studies have shown that the migration of peoples from malarial endemic regions to less or non-immune communities can lead to the serious threat of malaria reintroduction in malaria free-areas [7,8]. By 1985, an estimated 3.2 million Afghan refugees were living in Pakistan. Furthermore, subsequent US-military operations after September 11, 2001, incident triggered a substantial population displacement of the Afghan refugees to KPK, Pakistan [11,12]. Since that time, due to an open-border policy there has been continued back and forth migration of Afghan refugees across the border. The migration of Afghan refugees has overwhelmed the local public health system, resulted into ~24%–36% increase in malaria prevalence in bordering areas of Pakistan. The results from one report provided by the malaria control programs of Pakistan’s ministry of health have revealed a high prevalence of malaria among Afghan refugees in contrast to Pakistani residents in KPK province [13].

FATA area was a semi-autonomous territory running along the Pakistan-Afghanistan border until being merged with the adjoining KPK province. It is comprised of seven districts (previously called agencies). After September 11, 2001, incidents, these districts were a major theatre of militancy. Since 2001, Pakistan’s army launched 10 major operations against Taliban in these districts resulting in the movement of about two million IDPs, as hospitals, schools and homes were destroyed. Tribal districts are long neglected areas with poor living conditions, a lack of basic health necessities, limited access to vaccines, unequal distribution of economic resources, limited use of vector control measures. Most importantly, this is the case for approximately 60% of the residents. Currently, these districts have the high burden of different infectious diseases, due to the presence of a huge number of IDPs and the Afghan Refugees. In this situation, the population suffering from or most at risk of contracting malaria has significantly increased in the tribal districts as did the malaria parasite reservoir.

Moreover, since Pakistan’s independence (1947), the tribal district shave become the most impoverished and extremely underdeveloped areas in Pakistan with appalling social development indices [14]. Due to limited access to nearby local health care centers and/or less availability of antimicrobial drugs, malarial incidents are elevated in the area. Furthermore, heavy rainfall during the monsoon season, poor hygiene and rivers make this area very ideal for breeding of malarial parasites (Table 1). Therefore, for the proper detection and control of malaria in the tribal districts, obtaining and analyzing reliable data is essential.

**Table 1:** Summary of climatic data for the six study sites in KPK, Pakistan. Data include altitude, average yearly rainfall, population, community type and dominant ethnicity in the region. The sites are endemic for both malarial species (*P*. *vivax* and *P*. *falciparum*).

Location	Altitude	Average yearly rainfall (mm)	Average temperature (°C)	Population (inhabitants)	Community type	Dominant ethnicity
Orakzai District	1645	638	22	254,356	Rural	Pashtun
Kurram District	1705	785	20.6	619,553	Semi-urban	Pashtun
Hangu District	810	536	20.7	518,798	Rural	Pashtun
Bajaur District	870	738	23	1,093,684	Rural	Pashtun
Khyber District	1070	519	23	986, 973	Rural	Pashtun
Mohmand District	1450	422	25	466,984	Rural	Pashtun

In our previous study [14], we reported on the epidemiology and clinical burden of malaria in only one district (Orakzai) of the tribal area of KPK, Pakistan where the situation remains unchanged. However, in the current report, we studied the prevalence of malaria in six districts. Moreover, very limited research has been conducted to report household level characteristics associated with malaria, its prevalence, and clinical manifestations in all six tribal districts because of military conflicts in the past decades. It has been a challenge to carry out such studies due to the severity of military conflicts. However, the security situation is much better now. We report here the first detailed study of household level characteristics, epidemiology, and clinical burden of this war-torn region.

## Methods

### Ethics statement

Ethical approval for project activities was provided by Kohat University of Science and Technology and Quaid-i-Azam University, Islamabad (BEC-FBS-QAU-14). Written informed consent was obtained from the patients and their parents/guardians prior to recruitment.

### Study area and health infrastructure

**Fig 1:**
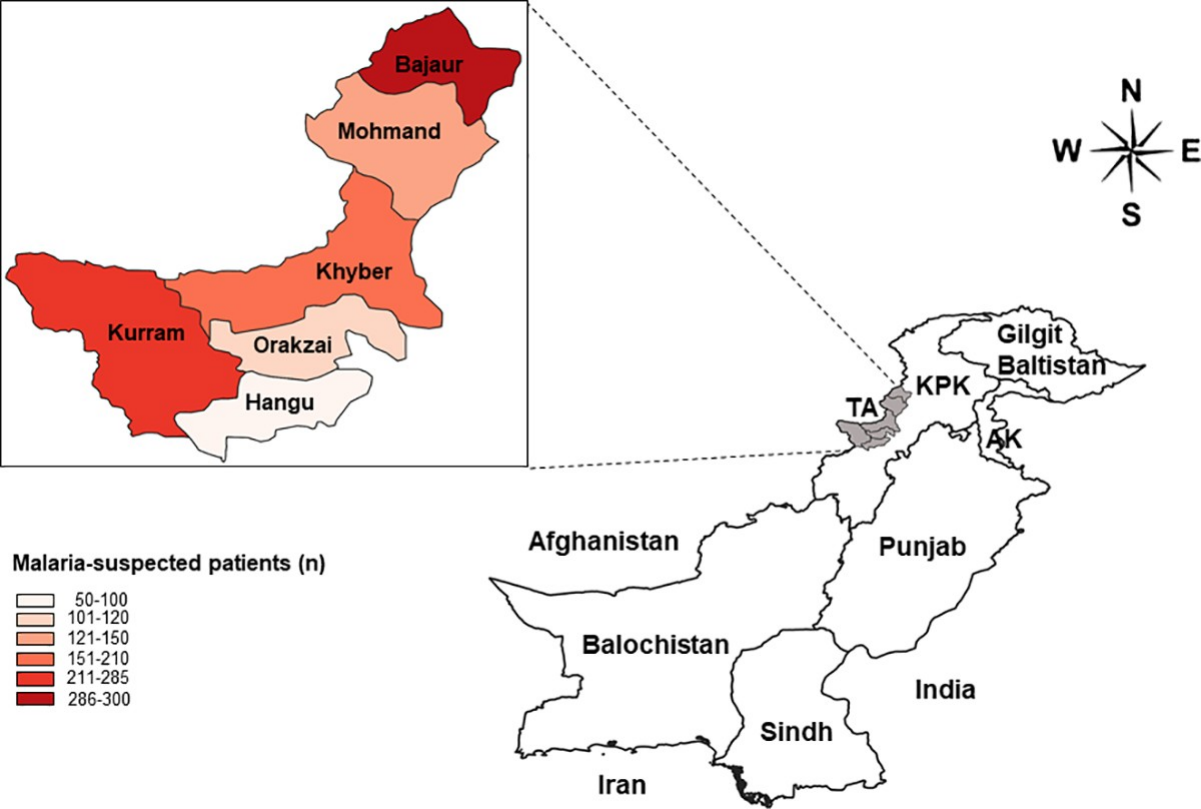
Geographical location of sample collection sites (six districts: shaded grey area) in KPK, Pakistan. **The enlarged box shows the prevalence of malaria in each district. KPK: Khyber Pakhtunkhwa, AK: Azad Kashmir, TA: Tribal Areas. The map in this figure was produced using ArcGIS version 10.4. Source of shapefile: United Nations Office for the Coordination of Humanitarian Affairs** https://data.humdata.org/dataset/pakistan-administrative-level-0-1-2-and-3-boundary-polygons-lines-and-central-places.

Tribal districts (Fig 1) in Pakistan are a long-neglected area with unequal distribution of economic resources, poor living conditions, lack of basic health facilities, limited use of vector control measures, and limited access to vaccines. Currently, tribal districts have the highest burden of different infectious diseases, due to the significant number of Afghan refugees and IDPs. The population suffering from or at risk of contracting malaria significantly increased in these districts as did the malaria parasite reservoir.

### Sample collection

This study was a retrospective case-control design and was carried out from March 2016 to December 2018 in the different hospitals of six tribal districts of KPK, Pakistan (Fig 1). The data were mined from respective health institutions including basic health units (BHU), community health centers (CHC), and district headquarter hospitals (DHH) in the six districts (District Bajaur, Mohmand, Khyber, Kurrum, Orakzai and Hangu). All adults and children presenting to different hospitals were screened for study eligibility. Patients presented with major malarial clinical features (headache, fever, dyspnea, cough, diarrhea, vomiting, abdominal pain, and convulsions) were considered for the study. Upon enrollment, a pre-tested interviewer-administered questionnaire was used to collect information on household characteristics, assets, education, use of insecticide-treated bed nets, repellent coils and fluids, malaria-related behavior and stagnant water bodies near homes. Moreover, demographic and clinical records were collected upon enrollment, and venous blood samples were collected for the routine measurement of hemoglobin, platelet count and further biochemical and molecular analyses. A total of 1,127 cases ranging in age from 1–60 years old were evaluated in this study. Pregnant women, bacterial or viral infections and other ailments that could explain the clinical presentation were excluded from this study. The control group was collected at the same sites as uncomplicated cases and used for statistical analyses. To eliminate the confounding effects of age, sex and locality, the control group was matched according to age, sex, and locality. Inclusion criteria for the study were: (1) hyperpyrexia, (2) respiratory problems, (3) impaired consciousness, (4) hypoglycaemia, (5) prostration (unable to sit), (6) anemia, (7) decompensated shock, (8) dark urine, (9) jaundice, (10) multiple convulsions, (11) hyperparasitaemia, and (12) multiple seizures. In severe malaria, the level of impaired consciousness was assessed by computing the Glasgow Coma Scale (GCS) score (<11) in adults or Blantyre Coma Scale Score (<3) in children.

To eliminate the risk of haemolysis in *P*. *vivax* infected cases, a screening test was performed for the detection of Glucose-6-phosphate dehydrogenase (G6PD) deficiency immediately after the diagnosis of malaria (15).

### Clinical assessment

After a detailed clinical investigation of patients, a standardized case report form was designed to compile the complete clinical data of each patient regarding their condition. In the case of children younger than five years, the history of the patient was gathered from their parents or relatives.

### Microbiological diagnosis

Baseline venous blood samples were taken for microscopy and nested polymerase chain reaction (nPCR) analysis. The initial diagnosis of *Plasmodium* spp. infection was made by thick or thin smears. Two slides were made from each patient’s blood and both thick and thin films were prepared on the slides in the respective laboratories. Giemsa-stained thick blood smears of patients were examined using Giemsa stain and the parasitemia was quantified independently by two skilled microscopists [14]. A thick smear was considered negative if no parasite was visible in at least 200 fields.

### Isolation of parasite DNA and molecular examination

For molecular examination, the parasite DNA was extracted from filter papers using a Qiagen DNA extraction kit (QIAGEN, Valencia, CA, USA), following the manufacturer’s protocol. Amplification of the small subunit ribosomal ribonucleic acid genes were carried out using nested PCR (nPCR) according to the methods described in our previous study [14]. Due to the absence of well-equipped laboratory facilities in tribal districts, biochemical analyses and nPCR were carried at the Kohat University of Science and Technology and Kohat Hospital.

### Statistical analysis

Statistical analysis was carried out using SPSS. Means and χ2 test of independence were calculated where applicable. In all the studied parameters, a p value of ≤ 0.05 was considered statistically significant.

## Results

### General characteristics of the study

Overall prevalence of malaria was 38% in our study. A total of 1,127 blood samples were collected from malaria-suspected patients residing in six tribal districts between March 2016 and December 2018. The median age of the patients was 22.5 years, of which 34% (n = 387 out of 1,127) were females and 66% (n = 740 out of 1,127) were males. Among 1,127 malaria-suspected patients, 78% (n = 882 out of 1,127) were diagnosed positive by the microscopic examination. This microscopic analysis detected 762 patients (68%) to be infected by *P*. *viax*, and 120 patients (11%) by *P*. *falciparum*. Patients infected by both *Plasmodium* species tended to less commonly have formal education and ownership of wealth indicators (e.g., fridge, TV set) was lower (Table 2). About 55% of the patients were using bed nets, while 31% stated the use of repellent coils and 20% cases stated the use of repellent fluids. Most importantly, 47% of cases stated there were stagnant water bodies at or near homes (Table 2). Moreover, we detected 6 (n = 6 out of 882, 0.7%) G6PD deficient patients, 4 of them males.

**Table 2:** Characteristics of 1,127 malaria-suspected patients from tribal districts of KPK, Pakistan.

Characteristic	All patients	No. (%) *P*. *vivax cases*, *n = 373*	No. (%) *P*. *falciparum* cases, n = 56	*p* value
No (%)	1127 (100)	762 (68.0)	120 (11.0)	-
Sex				
Males (n, %)	740 (66.0)	521 (68.0)	88 (0)	0.36
Females	387 (34.0)	241 (32.0)	32 (0)	0.25
**Origin (n, %)**				
Hangu District	102 (9.0)	61 (8.0)	11 (9.0)	
Kurram District	285 (25.0)	193 (25.0)	15 (12.5)	
Orakzai District	122 (11.0)	77 (10.0)	10 (8.0)	
Bajaur District	287 (25.0)	242 (32.0)	36 (30.0)	
Khyber District	206 (18.0)	108 (14.0)	23 (19.0)	
Mohmand District	125 (11.0)	81 (11.0)	25 (21.0)	
**Formal education (n, %)**				
None	493 (44.0)	358 (47.0)	44 (37.0)	
Incomplete (<10^th^ class)	315 (28.0)	223 (29.0)	31 (26.0)	
Completed (10^th^ class)	223 (20.0)	145 (19.0)	25 (21.0)	
Advance [(pre-)university]	96 (8.5)	36 (5.0)	20 (21.0)	
**Household characteristics (n, %)**				
Electricity	988 (88.0)	673 (88.0)	95 (79.0)	0.402
Electric fan	674 (60.0)	411 (54.0)	71 (59.0)	0.23
TV set	322 (29.0)	267 (35.0)	14 (12.0)	0.19
Fridge	127 (11.0)	71 (9.0)	15 (12.50)	0.11
Radio	1033 (92.0)	696 (91.0)	106 (88.0)	0.36
Stated use of aninsecticide-treated bednetsin preceding night	624 (55.0)	463 (61.0)	46 (38.0)	0.21
Stated use of repellent coils	347 (31.0)	154 (20.0)	38 (32.0)	0.31
Stated use of repellent fluids	224 (20.0)	188 (25.0)	9 (7.50)	0.56
Stated stagnant water bodies at or near home (n, **%**)	532 (47.0)	422 (55.0)	68 (57.0)	0.41

p value of ≤0.05 was considered statistically significant

The microscopy identified *Plasmodium*-positive patients were further analyzed by nPCR. In contrast, nPCR analysis detected 47% (n = 429 out of 882) *Plasmodium*-positive patients. This method detected 373 (87%) and 56 (13%) *P*. *vivax* and *P*. *falciparum* patients, respectively. Among these patients, the highest malaria cases were reported to be from the districts of Bajaur and Kurram. More importantly, average annual rainfall was highest in Kurrum (785 mm) and Bajaur (738 mm) districts, respectively (Table 1). There were no deaths reported in our study and3% of patients (n = 13 out of 429) were hospitalized for more than 3 days. 37% (n = 157 out of 429) patients infected by both *Plasmodium* species were children of the age group between 1 and 15 years old as shown in Table 3. We observed a high prevalence of *P*. *vivax* malaria in children (35%; n = 132 out of 373) and among late adolescents (between the age of 15–20). The incidence of both *Plasmodium* species gradually increased between the ages of 1–20.

**Table 3:** Patients’ history according to the malaria parasite species [18, 19].

Species			*P*. *vivax*			*P*. *falciparum*	
**Age groups**		<4 years	4–15 years	> 15 years	<4 years	4–15 years	> 15 years
**No. of subjects**		11 (3%)	121 (32%)	241 (65%)	4 (7%)	21 (38%)	31 (55%)
**Characteristics**							
Females		4 (1%)	25 (7%)	60 (16%)	2 (4%)	8 (14%)	13 (23%)
Males		7 (2%)	96 (26%)	181 (48.5%)	2 (4%)	13 (23%)	18 (32%)
Fever		11 (3%)	121 (32%)	241 (65%)	4 (7%)	23 (41%)	29 (52%)
Cough		8 (2%)	86 (23%)	109 (29%)	0	2 (4%)	0
Chills		6 (2%)	121 (32%)	218 (58%)	1 (2%)	6 (11%)	18 (32%)
Headache		5 (1%)	120 (32%)	231 (62%)	1 (2%)	8 (14%)	23 (41%)
Muscle pain		1 (0.27%)	104 (28%)	189 (51%)	0	15 (27%)	31 (55%)
Fatigue		1 (0.27%)	110 (29.5%)	186 (50%)	3 (5%)	14 (25%)	30 (54%)
Back pain		0	69 (18.5%)	143 (38%)	1 (2%)	12 (21%)	23 (41%)
Sweats		0	64 (17%)	180 (48%)	1 (2%)	4 (7%)	31 (55%)
Nausea		0	24 (6%)	89 (24%)	0	2 (4%)	12 (21%)
Vomiting		1 (0.27%)	9 (2%)	17 (5%)	2 (4%)	9 (16%)	6 (11%)
Diarrhoea		1 (0.27%)	4 (1%)	0	1 (2%)	1 (2%)	0
Concurrent illnesses							
Diabetes		0	0	4 (1%)	0	0	2 (4%)
Liver disease		0	0	3 (0.8%)	0	0	0

### Malaria diagnosis

The diagnosis was primarily made by microscopic examination. Among 1,127, 882patients (n = 882 out of 1,127; 78%) were identified having malaria, a conclusion drawn by microscopic examination. These (n = 882 out of 1,127; 78%) microscopically identified patients were further analyzed by nPCR analysis. In contrast, nPCR analysis detected 49% cases (n = 429 out of 882) to have contracted malaria. Moreover, in contrast to microscopic results, nPCR analysis detected 87% *vivax* malaria cases (n = 373 out of 429) and 13%*falciparum* malaria cases (n = 56 out of 429). A significant level of inconsistency between microscopic and nPCR diagnosis was observed. According to comparative studies, nPCRismore sensitive and reliable for *Plasmodium* diagnosis then other techniques [14–17]. Because of the noticeable discrepancies between microscopy and nPCR detections, we decided to rely on nPCR diagnosis.

### Clinical characteristics

We observed a greater prevalence of *Plasmodium* cases in males (74.0%) than females (26.0%). Most of the male (n = 284; 76%) and female (n = 89; 24%) cases had *vivax* malaria while 59.0% (33 of 56) of malesand41% (23 of 56) of female cases had contracted *falciparum* malaria as shown in Table 3.Subsequent to admission the reported symptoms and signs were not different regarding parasite species although fatigue was commonly observed by *falciparum* malaria cases as compared to patients with *vivax* malaria, and fever and cough (mostly observed in *vivax* malaria patients) were commonly observed in all cases reported (Table 3). We observed 2% (9 of 429) of the reported cases to have existing co-morbidities (Table 3). The clinical and biochemical results of cases with *P*. *falciparum* and *P*. *vivax* malaria revealed the majority of cases showed an increased proportion of hyperpyrexia (core body temperature >40°C) (Table 4). For the treatment of *P*. *vivax* malaria, chloroquine and primaquine were used. To treat *P*. *falciparum* malaria, sulfadoxine/pyrimethamine (on day 1) and Artesunate (for three days) were used according to the patient’s age. To treat severe malaria, artesunate or quinine were administered.

**Table 4:** Comparison of complication rates in *P*. *vivax* versus *P*. *falciparum* infection, Tribal districts, KPK province, Pakistan, 2016–2018 [18, 19].

		Frequency (%)					
WHO		*P*. *vivax* <4 years 4–15 years >15 years			*P*. *falciparum* <4 years 4–15 years >15 years		
Complications	Case definition						
Anemia	A Hg concentration <5 g/dL in children or <7 g/dL in adults together with a parasite count >10,000/μL	4 (1%)	35 (9%)	28 (18%)	0	3 (5%)	1 (2%)
Decompensated shock	Systolic blood pressure <70 mm Hg in children and <80 mm Hg in adults with evidence of impaired perfusion (cool peripheries or prolonged capillary refill)	1 (0.2%)	19 (5%)	13 (3%)	1 (2%)	2 (4%)	4 (7%)
Hyperpyrexia	Core body temperature >40°C	4 (1%)	52 (14%)	49 (13%)	2 (4%)	7 (13%)	3 (5%)
Hyperparasitaemia	*P*. *falciparum* parasitaemia>10% of total red cells or lower parasitaemia in case of *P*. *vivax*	3 (0.8)	16 (4%)	23 (6%)	1 (2%)	2 (4%)	4 (7%)
Hypoglycemia	Blood or plasma glucose concentration <2.2 mM (<40 mg/dL)	2 (0.5)	20 (5%)	13 (3%)	0	3 (5%)	7 (13%)
Jaundice	Plasma or serum bilirubin >50 mM (>3.0 mg/dL) and parasite count >100, 000/μL	0	11 (3%)	35 (9%)	1 (2%)	3 (5%)	4 (7%)
Multiple convulsions	Generalized seizures (particularly in children), twitching of a digit, repetitive jerky eye movements with deviation, or increased salivation	1 (0.2%)	17 (5%)	11 (3%)	1 (2%)	4 (7%)	3 (5%)
Petechia (%, n)	1–2 mm red or purple spot on the skin	2 (0.5%)	9 (2%)	5 (1%)	0	0	0

WHO, World Health Organization; CI, Confidence Interval; Hg, hemoglobin.

## Discussion

In this study from the tribal districts of KPK in Pakistan, almost 87% of malaria episodes were due to *P*. *vivax*. Overall, both species causing malaria showed a pronounced clinical manifestation. Despite the highest mortality of malarial infections in Pakistan, the preventive approaches in far-flung regions are still insufficient. Our study shows the emerging malarial incidents in the tribal districts especially in distal areas. This is possibly due to unawareness, no utilization of insecticides treated bed-nets and low availability of malarial drugs to these areas. Additionally, due to emergency law-and-order situation in tribal districts accelerate limited health care provision [14–19].

Pakistan experiences both *P*. *vivax* (78%) and *P*. *falciparum* (21%) according to WHO report. The diversity in prevalence and species distribution of malaria-causing parasites varies in different parts of the country and unfortunately it has not been fully described [20]. However, due to insufficient or imprecise data availability it is hard to estimate the prevalence of dominant *Plasmodium* species or the type of infection in the FATA region of Pakistan [15]. We sampled within the transmission season (the *P*. *vivax* transmission season peaks between April and September, while the *P*. *falciparum* peaks between August and December) [14]. However, regional variations may happen at peak times. One study conducted in the hilly region of tribal districts reported the highest *P*. *vivax* cases and lowest *P*. *falciparum* cases in March and the opposite pattern in October [14]. These effects should be taken into account when assessing relatively small reported differences in the prevalence of malaria and in the proportion of *P*. *vivax* and *P*. *falciparum* cases among regions [16].

In our study, a significant level of discrepancy between microscopic and nPCR diagnosis was observed. In microscopy, the possibility of misdiagnosis of Plasmodium species is high, particularly for low parasitemia, mixed infections, and when only ring forms are seen. Previous comparative reports have shown that microscopic diagnosis does not reliably distinguish *Plasmodium* species in the endemic areas and declared nPCR to be more reliable and sensitive for malaria diagnosis than other techniques [14–16].

Anopheles in the area adapting to breed in waste water (*An*. *culicifacies* breeds in drains filled with waste water, however, *An*. *stephensi* breeds mainly in clean/clear water containers, and *An*. *fluviatilis* in streams) [21,22,23]. As *P*. *vivax* is highly prevalent in hilly areas, similarly, all six studied districts are covered with hills, making these districts appropriate for the breeding of parasites [16]. Moreover, high annual rainfall in the studied districts also creates breeding sites for female mosquitoes to lay eggs. We were unable to find any *P*. *malariae* and *P*.*ovale* infections in our samples. This finding also agreed with the fact that these two species had been negligible in this region [14,24,25].

Laboratory and clinical findings revealed that hyperpyrexia and anemia were more frequent in our studied patients (Table 4). Similar results of prevalent anemia have also been reported previously in cases infected by *P*. *vivax* [24,26]. Furthermore, 13% of cases infected by both species were found to be with jaundice as reported in previous reports [14,27].

In this study, we found children were excessively susceptible to malaria. We observed 37% (n = 157) of malaria cases were children (≤15 years old). Similar findings of prevalent malaria in children have also been reported in our previous report (14) as well as in other studies [24,28]. This may possibly be attributed to poor yet-to-develop immunity against malaria. We observed higher number of cases in the Bajaur district, followed by Kurram and Khyber districts and a high proportion of cases attributed to *P*. *vivax* as reported in previous studies [14–16]. Furthermore, cross-border migration may have contributed to the maintenance or surge of malaria in this region [23]. After the year 2000, refugees from Afghanistan fled across the border into tribal districts and KPK Province. This influx of a potentially more malaria-susceptible population may have overwhelmed the public health system, leading to greater disease [14–16]. The movement of Afghan refugees into Pakistan’s Balochistan province and Iran was estimated to result in a 24–36% increase in the number of malaria cases [28–29]. Recent internal displacement may also be contributing to the high prevalence of malaria. Due to Pakistan’s continuous military operations against militants since 2001–2015, inhabitants of several areas in the tribal districts fled war conditions to settle in adjoining districts. These large movements of vulnerable populations may have altered the distribution of malaria and malaria-susceptible people in the country, contributing to epidemics [14–16].

The vast majority (more than 60%) of residents of tribal districts are predominately socio-economically underprivileged along with the lack of public health infrastructure. Approximately 55% of households owned a bed net (624/1127) in this study. The age of the participants was an important factor when bed net usage was taken into consideration. Bed net usage was higher in children under the age of 2 years and fell among late teenagers. A higher level of education, possession of TV sets, use of bed nets, use of repellent fluids, and a fridge were all associated with protection from malaria and these factors also serve as a marker of wealth. Therefore, this pattern may have influence on the awareness and knowledge of malaria and hence on the clinical picture.

Nevertheless, our study has its own limitations. Our study failed to carry out the rapid diagnostic test (RDT) for all patients. Therefore, some patients may have been overlooked who could be positive for malaria. In our study, noticeable discrepancies between microscopy and nPCR were observed. However, like other diagnostic studies, we relied on nPCR diagnosis.

## Conclusions

Despite some limitations, this study is the first detailed report on the households, epidemiological situation and clinical analysis regarding this most neglected tribal region. Moreover, children (1–15 years) and late teenagers (15–20 years) were the most susceptible population to malaria in the area. Our findings also highlight that malaria remains a disease of the poor. An increased investment in socio-economic development, strong health infrastructure, and malaria education are key interventions to reduce malaria in tribal districts.

## Supporting information

S1 Fig Excel spreadsheet containing the underlying numerical data for Fig 1. (XLSX)
